# Q&A on working part-time in academia

**DOI:** 10.1038/s42004-023-00957-8

**Published:** 2023-07-20

## Abstract

Dr Zoe Schnepp and Professor David Smith share their experiences of working part-time in academia, discussing some of the benefits and challenges, and offering advice to those who may be seeking to work part-time.

Zoe Schnepp is Associate Professor of Chemistry at University of Birmingham. Her research focuses on biomass-derived materials and particularly the way that iron is able to convert amorphous carbon from biomass into graphitic carbon. Zoe also leads the ChemBAM project (www.chembam.com), which aims to bring chemistry to life through fun and accessible experiments and activities. The ChemBAM team won the 2023 Royal Society of Chemistry Inclusion and Diversity Prize.

**Figure:**
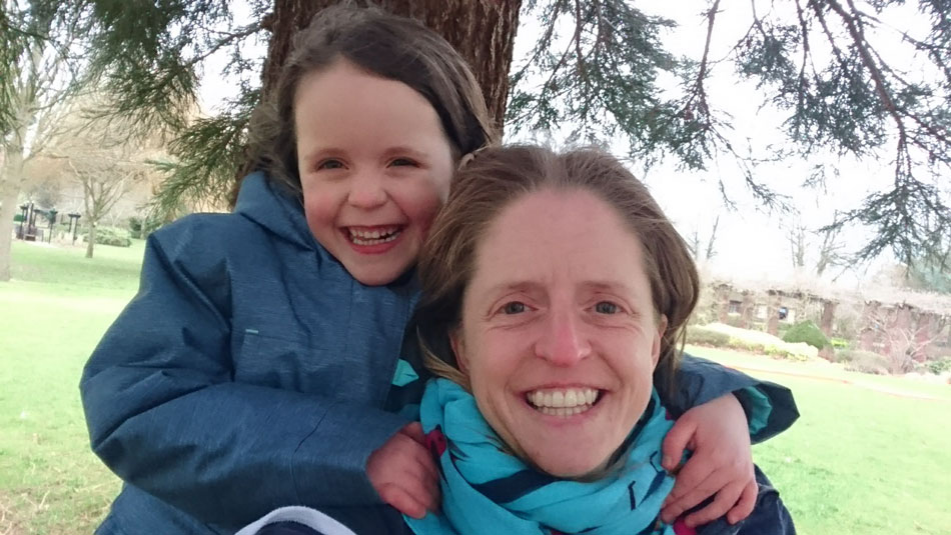
Zoe Schnepp

David Smith is Professor of Chemistry at University of York where he carries out research into self-assembling molecular materials. In 2022, he was awarded the Royal Society of Chemistry ‘Tilden Prize’ in recognition of his research. He also received the 2022 Society of Chemical Industry’s ‘Science for Society Award’ for chemical education and outreach. Dave has written and lectured widely on wide-ranging aspects of inclusion and diversity in modern science, and was recognized by Chemical and Engineering News as an ‘LGBT + Trailblazer’. In memory of his husband, his love of food and cooking led him to write the award-winning cookbook/memoir, ‘Tw-Eat Together’.

**Figure:**
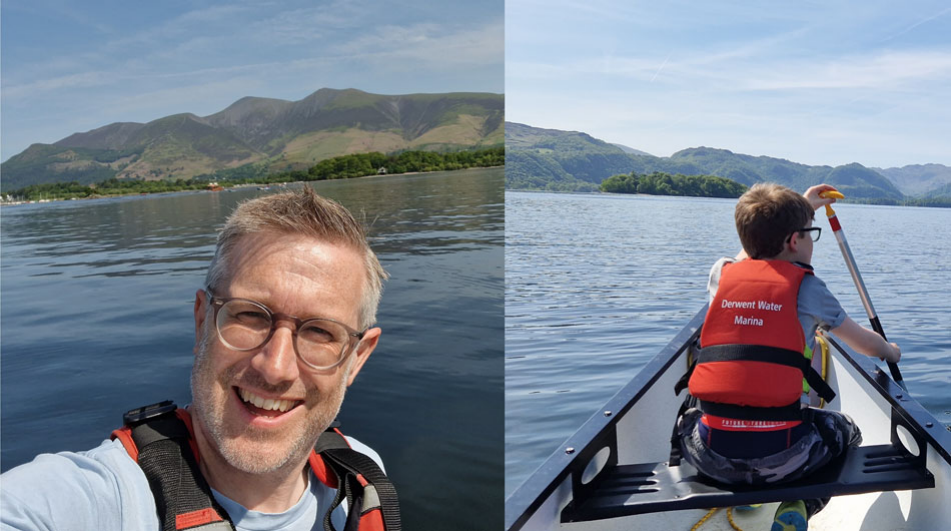
David K. Smith

When and how did you decide to start working part-time?

**David**: I first worked part-time during the year after my husband and I adopted our son in 2014. This allowed me to spend more time at home, helping to build the life of our new family, while my husband was on full parental leave. Having returned to full-time working, I then went back to part-time working as my husband became very ill in 2018 with rejection of his lung transplant. I needed more time to do things at home. After his death in 2019, I decreased my hours further to have enough time to manage being a single parent to a child with additional needs. Currently, I am working 80%. Essentially, at this level, all of my time is spent either working, or being a parent.

**Zoe**: I started working 80% (4 days a week) when I went back to work after my first daughter was born in 2016. I was happy with her going to nursery but wanted to have more time with her than two days each weekend. After my first daughter started school, I realized how difficult it was going to be to manage school holidays without any family living nearby, so I moved to 70% full-time equivalent on a flexi-part-time contract. I work 4 days a week during term time and take most of the school holidays off.

What support does your department/institution offer for part-time working?

**David**: My department was very supportive of part-time working. They offer a part-time assurance that part-time working should always be seen as a possibility, and furthermore, that the choice is a reversible one, and that moving back to full-time hours would be possible (apart from in exceptional circumstances). This made the decision to work part-time easier. There are also quite a significant number of people here, including very successful academics, working part-time. These local role models mean that part-time working is not seen as a negative.

Most importantly, my department facilitated a very flexi-style part-time working pattern. This means that I don’t have 1 day off a week, but rather I actually work full time during the academic term (which often aligns with school term times). I ‘save up’ all of my days off and use them in school holidays, which, for me, are the hardest bits of childcare to find any cover for. This working pattern has been hugely beneficial — without my department formally facilitating it, I’m not sure I could have carried on being an academic. A standard academic contract here in the UK has a total of ca. 37 vacation days (including all national holidays), whereas a child has 72 days off school — that’s a big (35 day) gap for a single parent to fill.

**Zoe**: My department has been very supportive of my part-time working. Both transitions to part-time working were started with a 1 year trial, so I didn’t have to make a permanent change to my contract until I was certain it was the right decision for me. My department has also allowed me a lot of flexibility in planning my time. Rather than taking the whole summer holiday off (like a traditional annualized contract), I work a few days over the summer to keep up with my research group. Then I use those few extra holiday days as wellbeing days during termtime. This has been invaluable for ensuring I occasionally have some time to myself!

What have been the biggest benefits for you?

**David**: The biggest benefit is being able to walk out of the department during school holidays, go and do activities with my son, and not feel guilty. If the e-mails pile up, so be it. Essentially, dropping hours removes the guilt associated with the concept that you should always be working on something, which is sadly so ingrained into academic life. This has improved the quality (and quantity) of the time I have had to spend with my son. I have been able to do things with him, and give him the time, that simply would not have been possible without it. The alternative would have been round-the-clock childcare, but we adopted a child because we wanted to be parents, and for him to experience a loving family. After my husband (his Pop) died, I couldn’t just have put him in childcare every day.

**Zoe**: I have really valued being able to commit more time to my family. As David already mentioned, there’s a culture in academia of always feeling that you should be working. Making the decision to take a pay cut and go part-time means I feel no guilt when I have days off with my daughters on Fridays or in school holidays. My husband also works flexi part-time, so it is giving us time for some great adventures during school holidays!

What challenges do you most commonly face as a result of working part-time?

**David**: Prior to going part-time, I probably worked about 55 h a week, even though my contract was for 37.5 h per week. Once I shifted to 80% time, I was officially being paid to work 30 h per week. I became much more conscious of the hours I was working, and I had calculated the amount of work I could actually offer very precisely to fit around my family responsibilities. Stopping doing that unpaid extra academic labour means that even though my formal workload got adjusted a bit, it never feels like enough — I am always struggling to get the things that I should be doing, or used to do, done, let alone the things that I would really like to do.

I was once asked, “If you worked 55 h a week before, why, at 80% contract are you not happy to work 44 h a week?” It doesn’t work like that! If I’m only being paid for 30 h, and every hour is absolutely precious and precisely calculated to make my family work, then 30 h is all I can give. The problem is not with me being unprepared to do unpaid overtime, the problem is with academia for routinely expecting it.

**Zoe**: The biggest challenge is that I haven’t dropped from 100%! I’ve always tried to have a good work–life balance, but before I had children I definitely worked more than the standard 37.5 h week. When I went part-time and took the pay cut, I was much more focused on working within my contracted hours. I’ve found it really hard to manage my own expectations of how much I can reasonably achieve in a year. I’ve chosen to work fewer hours but I’m still ambitious. It’s taking me some time to accept that I can’t do everything!

Could changes to your workplace culture or practices help alleviate some of these challenges?

**David**: I think the part-time assurance and formal adjustments to the workload model in my own department both helped me, as did the fairly widespread culture and acceptance of part-time working. I can imagine that if you were in a department without those, things would be really tough.

More widely, I wish academia was more focussed on the quality of what people do, rather than the quantity. If someone working part-time runs a smaller research group, travels less, and publishes fewer papers, that shouldn’t matter, as long as the quality of their mentorship, presentations and research are excellent. (Indeed, my own University explicitly now promotes people based on meeting quality markers, with typical quantities adjusted for part-time working).

**Zoe**: I think wider visibility of role models will really help. When I went part-time, I was the only academic in my department on that type of contract. I didn’t really have a role model of a successful part-time academic. I saw a talk by a Professor from another institution and, alongside her research, she described the challenges she’d faced by being part-time for most of her career. It was really inspiring and heartening to see someone balance part-time work with a successful academic career. I’d like to see more stories like that and more diversity in what success looks like in academia, rather than success being a measure of how fast someone gets to a full professorship!

I would also like to echo what David said about quality. Early in my career I was encouraged to push for more funding and a larger research group. There was very much a message that success meant a large research group and lots of publications. It’s taken me a long time to realize that focussing on a small but high-quality research effort is equally valid.

What advice would you offer to other academics seeking to work part-time?

**David**: Be prepared to be surprised to discover how many unpaid hours you were previously doing, and how difficult it is to get rid of them. Be ruthless when discussing your workload with management, and be very honest with yourself about what you can really achieve working part-time.

Make sure that when you are not working, you are really not working. Don’t be half on e-mail, half thinking about a grant, and half trying to look after your family. Ringfence your time and use it, benefit from it, revel in it. Make sure that when you look back, you can think about all the better memories you have because of things you could do when you were part-time working, rather than looking back and regretting what you didn’t do at work.

**Zoe**: I would definitely agree with David’s comment about being ruthless. Academia will take as much time as you give it. It’s hard to turn down requests but you have to make sure that the time you spend working is used on things that you really value. I also agree that you have to set boundaries and work only when you are scheduled to work. I’d add though that it’s difficult to be efficient all of the time. I find that because of my limited hours, I’m really hard on myself if I have a bad day at work. If you can find a way to be kind to yourself and accept that you can’t be a super-academic and a super-parent all of the time, then life will probably be a lot easier!

Do you plan to go back to full-time working one day?

**David:** Although my son is getting older (he’s now 10), he has additional needs, so I suspect I may be part-time working for some time to come. I may one day go back to full-time working, but I would also consider using the extra time for other pursuits. I’ve enjoyed all the extra adventures with my son that part-time working has enabled, and wonder what else I could achieve alongside my day job.

**Zoe**: Definitely not! My youngest daughter starts school in September and I’m really looking forward to having a day a week to focus on hobbies. I actually find that taking some time away from work can help me with the creative side of being a scientist. Academia is so intense and I often find the week has flown by without having a minute to step back and think.

*This interview was conducted by the editors of Communications Chemistry*.

